# Modeling the effect of longitudinal markers on left-truncated time-to-event outcomes in twin studies

**DOI:** 10.1177/09622802251383643

**Published:** 2025-10-14

**Authors:** Annah Muli, Mar Rodriguez-Girondo, Jeanine Houwing-Duistermaat

**Affiliations:** 1 Department of Statistics, 120986 University of Leeds, UK; 2 Leeds Institute of Clinical Trials Research, University of Leeds, UK; 3 Department of Biomedical Data Sciences, Leiden University Medical Center, The Netherlands; 4 Department of Mathematics, Radboud University Nijmegen, The Netherlands

**Keywords:** Joint model, delayed entry, regression–calibration, shared frailty model, longitudinal data, twins

## Abstract

The identification of biomarkers for disease onset in longitudinal studies necessitates precise estimation of the association between longitudinal markers and survival outcomes. Currently, methods for estimating these associations in the context of left-truncated and clustered survival outcomes are lacking. In this study, we propose a novel model tailored to this scenario and develop several estimation methods: last observation carried forward, regression calibration, and a two-stage likelihood approach for joint modeling of longitudinal and survival processes. Simulation results indicate that the last observation carried forward method performs well only with a dense grid and no marker measurement error. For less dense grids and low measurement error, regression calibration approaches are preferred. Joint modeling approaches outperform calibration methods in the presence of measurement error, although they may suffer from numerical instability. In cases of numerical instability, calibration methods might be a good alternative. We applied these methodologies to the TwinsUK data to estimate the effect of bone mineral density (BMD) as a longitudinal marker on fracture incidence in 766 elderly females, 138 of whom experienced a fracture. The survival model included a shared gamma-distributed frailty to account for correlation between the times to fracture of twin pairs. Estimates obtained using calibration and joint modeling approaches indicated a larger BMD effect compared to the last observation carried forward method, likely due to the irregular BMD measurement process and minimal measurement error. Overall, our methods offer valuable tools for modeling the effect of a longitudinal marker on survival outcomes in complex designs.

## 1. Introduction

Modeling the relationship between a longitudinal marker and a time-to-event outcome is a popular research area. To date, most of this research has focused on modeling data from independent subjects (singletons), using time-in-study (follow-up time) as the primary time scale. For many diseases, however, age is the natural time scale, which results in left truncation of the time-to-event outcome. Additionally, longitudinal data collection often follows complex designs. Twin studies, as one of the most prominent sources of high-quality longitudinal data,^1,2^ present unique challenges due to their clustered nature. This paper focuses on estimating the association between a longitudinal marker and a left-truncated survival outcome in twin studies. Currently, no method exists to handle this type of data.

A common approach to model dependence of clustered event times is by introducing a cluster-specific random effect—the shared frailty model.^3–6^ The survival times are assumed to be conditionally independent given the shared (common) frailty. The gamma distribution has commonly been considered for the frailties because of mathematical convenience, since it typically produces a tractable marginal likelihood function for the parameters after integration. The literature on joint models for a longitudinal marker and a survival outcome in paired data is limited, and currently used models lack flexibility. Specifically, in the models of Ratcliffe et al.,^7^ and Brilleman et al.,^8^ the correlation between the survival times of cluster members is solely modeled by the normally distributed shared effects of the survival and longitudinal outcomes. This model can be fitted using the available statistical software (e.g., VAJointSurv,^9^ INLAjoint^10^ packages in R and gsem,^11^ and merlin^12^ in Stata). In this paper, we propose a more general model that allows for additional correlation by including a gamma-distributed frailty term in the survival model. The R package Frailtypack^13^ allows for additional correlation in the survival model. However, it can fit either a joint longitudinal and clustered survival model without delayed entry or a left-truncated clustered survival data with time-invariant markers.

Estimation of the parameters of joint models by maximum likelihood is time-consuming due to the necessary numerical integration of the normally distributed shared random effects in the survival model.^14^ Using a novel two-stage joint likelihood approach, we propose to jointly estimate the shared random effect of the longitudinal marker and the frailty term of the survival process, preceded by the plug-in of the best linear unbiased predictions (BLUP) estimates for the individual-specific random effects of the longitudinal marker.

Furthermore, for estimation of the relationship between the longitudinal marker and the survival outcome, we compare the performance of this new joint modeling (JM) approach with two classical and simpler approaches adapted to clustered and left-truncated data, namely the last observation carried forward (LOCF) approach and regression calibration techniques. In the context of independent data, and under the strong assumption that the longitudinal process only jumps at observed time points and remains constant between two consecutive observation points, the LOCF approach has been proposed for modeling the relationship between the marker and the time-to-event outcome in singletons. This method can be easily extended to clustered data by using shared frailty models, which can be fitted using available statistical packages (e.g., the coxph function in the survival package^15^ and the frailtypack^13^ package in R). However, the adjustment to left-truncation is notably more elaborated for clustered data than for the singleton case.

Methods have been developed for shared-frailty models, but are restricted to time-fixed covariates. Hence, there is no software available to estimate the effect of a longitudinal marker on a time-to-event outcome by the LOCF approach on clustered data with delayed entry. Here, we extend the method for time-fixed covariates to the context of longitudinal markers. Often, the assumption of constant values within observation intervals cannot be made. For such cases, regression calibration methods have been proposed for singletons.^16–22^ These are two-step approaches consisting of first fitting a mixed model to obtain estimates of the longitudinal marker. These estimated values can then be used in a second step as if they had been observed in the LOCF-based approach. Here, we extend regression calibration methods to the context of frailty models with delayed entry, which requires a more complex first step involving a linear mixed model with extra random effects to model the correlation between the longitudinal outcomes of cluster members. Using an intensive simulation study, we provide specific recommendations for estimation methods, taking into account the underlying model, the time gaps between longitudinal observations, and the level of measurement error.

As a data example, we consider bone mineral density (BMD), repeatedly measured over time, and the risk of fracture for twins from the TwinsUK registry. In a previous paper, we estimated the probability of a fracture in the next time period given current age and BMD using shared frailty models.^23^ For such a question, follow-up time is the natural underlying time variable. Here, we are interested in modeling the effect of BMD on fracture incidence. As with many other aging-related diseases, for such a model, age is the natural time scale, which, in combination with twins entering the registry at different ages (delayed entry), results in left truncation of the time-to-event outcome.

The contributions of this paper are threefold. Firstly, it presents a novel statistical model for the analysis of longitudinal and survival data in twins. Secondly, it introduces a new set of estimation methods, each offering increased flexibility and computational complexity, all adapted to deal with left-truncated survival data. Thirdly, it provides users with guidance based on simulation results, advising on the choice of the estimation method according to factors such as observation grid density and measurement error.

The rest of this paper is laid out as follows: in the second section, we formalize the problem and propose novel approaches, in the third section, we study the performance of the methods via simulations, in the fourth section we present the results of the analysis of the TwinsUK dataset to estimate the effect of BMD on age-specific fracture incidence using our novel approaches. Lastly, the fifth section provides the discussion and conclusions.

## 2. Methods

### 2.1. Notation and model formulation

We are interested in modeling the relationship between a longitudinal marker and survival times in twin pairs. Let N be the number of twin pairs. For twin j of pair i , let $T_{ij}$ be the random survival time of interest and $M_{ij}(t)$ be the longitudinal marker. Let $x_{ij}$ be a column vector of individual-specific time-invariant covariates for the survival time $T_{ij}$ and let $\gamma =(\gamma_{1},\gamma_{2},\ldots ,\gamma_{p})$ be a parameter vector for these time-invariant covariates. Let the frailty $v_{i}$ represent the unmeasured shared effects for twin pair i that have an effect on their survival times $T_{i1}$ and $T_{i2}$ . We propose a gamma distribution with mean one and variance θ for its distribution. Furthermore, not all $T_{ij}$ will be observed. So, define a second random variable $C_{ij}$ independent of $T_{ij}$ , which represents the censoring process.

Depending on the available amount of information on M(t) , we will model it either nonparametrically or by using the following random effects model:

(1) $$M_{ij}(t)=G_{ij}(t)+u_{i}$$

with $u_{i}$ zero mean and independent normally distributed random variables with variance $\sigma_{u}^{2}$ , which represents the deviation of the twin profile from the population profile $G_{ij}(t)$ . In case there are many observations $G_{ij}(t)$ might be modeled with a flexible fixed and random effects structure. In this paper, the following linear mixed model for $G_{ij}(t)$ is considered:

(2) $$G_{ij}(t)=\beta_{0}+\beta_{1}t+b_{ij0}+b_{ij1}t$$

where the vector $\left(b_{ij0},b_{ij1}\right)$ follows a normal distribution with zero mean and covariance structure $\Sigma_{b}$ . Here, $b_{ij0}$ represents the deviation of twin j from pair i of the population mean $\beta_{0}$ , and $b_{ij1}$ represents the deviation of the slope of time for twin j from pair i of the population slope $\beta_{1}$ .

Typically, $M_{ij}(t)$ is observed only at specific time points with a (small) error. For twin j of pair i , let the index k run over the subject-specific grid of time points $k\in \{0,\ldots ,K_{ij}-1\}$ , where $K_{ij}$ is the number of time points for this subject. Now, define the random variable $Y_{ijk}$ as a random perturbation of $M_{ij}(t_{k})$ at time point $t_{k}$ . Thus, the following relationship between $Y_{ijk}$ and $M(t_{k})$ is proposed:

(3) $$Y_{ijk}=M_{ij}(t_{k})+\varepsilon_{ijk},\;k=0,\ldots ,K_{ij}-1,\;j=1,2,\;i=1,\ldots ,N$$

where $\varepsilon_{ijk}$ are mutually independent normally distributed random variables with zero mean and variance $\sigma_{e}^{2}$ . Note that for $\sigma_{e}^{2}\to 0$ , $Y_{ijk}\approx M_{ij}(t_{k})$ .

Now, to model the relationship between M(t) and T , we need to consider two situations. Firstly, M(t) is considered exogenous if its value at any given time is not influenced by past events. We can operationalize the concept of exogeneity in our context, assuming that M(t) has a direct effect on T and that there are no unobserved confounders.^24^ For example, if M(t) represents air pollution levels over time and T denotes the onset of disease, then $M(t_{k})$ is as an exogenous variable. Secondly, M(t) is considered endogenous when its current value is affected by past events. This dependence naturally arises through unobserved confounding. Consequently, we define a longitudinal marker M(t) as endogenous if it affects T while also being influenced by unobserved variables that affect both M(t) and T . Many biomarkers follow this model. For example, unobserved genetic factors influence the biomarker and the survival times of the two twins.

**Figure 1.**
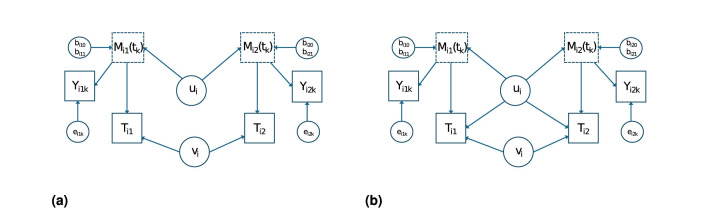
Model for the relationship between the marker profile M(t) and the survival time T for twins. (a) M(t) exogenous and (b) M(t) endogenous. When the variance of $e_{ijk}$ is small, $M_{ij}(t_{k})$ can be replaced with $Y_{ijk}$ .

Firstly, we consider the case that M(t) *an exogenous variable*. The model is depicted in Figure 1(a). Since there are no unobserved confounders for the relationship between M(t) and T , the following model for the conditional hazard function given the frailty $v_{i}$ , time invariant covariates $x_{ij}$ and the covariate trajectory $M_{ij}(t)$ , $h_{ij}(t|v_{i},x_{ij},M_{ij}(t))$ , can be proposed:

(4) $$h_{ij}(t|v_{i},x_{ij},M_{ij}(t))=h_{0}(t)v_{i}\exp\;\{\gamma x_{ij}+\alpha M_{ij}(t)\}$$

where the parameter α represents the effect of the variable $M_{ij}(t)$ on $T_{ij}$ . The function $h_{0}(t)$ is the baseline hazard, that is, the hazard when $M_{ij}(t)=0$ and $x_{ij}=0$ . In this paper, we consider parametric hazard functions with parameters ξ . For time points $t_{k}$ and small $\sigma_{e}^{2}$ , model (4) reduces to

$$h_{ij}(t_{k}|v_{i},x_{ij},Y_{ijk})=h_{0}(t)v_{i}\exp\;\{\gamma x_{ij}+\alpha Y_{ijk}\}$$

Secondly, for M(t) *an endogenous variable*, we assume that the random twin effect $u_{i}$ representing the shared effects for the two twins for the marker patterns $M_{ij}(t)$ is also shared with the random time variables $T_{ij}$ . Examples of twin pair shared effects are genetic and environmental factors. In this model, the frailty $v_{i}$ models the additional covariance between $T_{i1}$ and $T_{i2}$ , and $b_{ij}$ models the correlation of the $M_{ij}(t)$ and $M_{ij}(t^{\prime })$ for person j and $t\neq t^{\prime }$ . Figure 1(b) shows this model. The formula for this model is

(5) $$\begin{array}{rl} M_{ij}(t) & =G_{ij}(t)+u_{i}=\beta_{0}+\beta_{1}t+b_{ij0}+b_{ij1}t+u_{i}\\ h_{ij}(t|u_{i},v_{i},x_{ij}) & =h_{0}(t)v_{i}\exp\;\{\gamma x_{ij}+\alpha (G_{ij}(t)+u_{i})\} \end{array}$$

Note that by including the frailty $v_{i}$ this model is more general than the models of Ratcliffe et al.^7^ and Brilleman et al.^8^

### 2.2. Estimation of parameters

Let $t_{ij}$ be the observed survival time given in age scale and $t_{ij0}$ be the age of enrollment to the study for person j of twin pair i,j=1,2 . An individual is only included in the study if $t_{ij}>t_{ij0}$ . Note that if the enrollment times $t_{ij0}$ differ across subjects, we have delayed entry, and this needs to be accounted for when estimating the parameters. Furthermore, the observed time $t_{ij}$ of person j of twin pair i can be either censored or a time at which the event of interest has occurred. Let $\delta_{ij}$ be the event indicator, that is, $\delta_{ij}=1$ if $t_{ij}$ is an event time, that is, $T_{ij}\leq C_{ij}$ and $\delta_{ij}=0$ , that is, $T_{ij}>C_{ij}$ otherwise. Furthermore, let $y_{ijk}$ be the observed value of the random variable $Y_{ijk}$ , which is a perturbation of $M_{ij}(t_{k})$ with $k=0,\ldots ,K_{ij}-1$ , the grid of observed time points for person j of twin pair i . We assume that $t_{K_{ij}-1}\leq t_{ij}$ . In the next paragraphs, we will describe the estimation procedures for $M_{ij}(t)$ exogenous and a small error variance $\sigma_{e}^{2}$ , and for $M_{ij}(t)$ endogenous.

#### 2.2.1. Estimation of α by LOCF

When it can be assumed that the observations $y_{ijk}$ do not vary between consecutive time points $t_{k}$ and $t_{k+1}$ and the measurement error $\sigma_{e}$ is relatively small, we have $Y_{ij}(t_{k})\approx M(t_{k})\approx M(t)$ for $t_{k}\leq t<t_{k+1}$ and use $Y_{ijk}$ instead of M(t) in the survival model. The variation in a biomarker between consecutive time points depends on both the biomarker’s trajectory and the density of the observation grid, where denser grids make this assumption more plausible. A small measurement error is realistic for precise biomarkers such as body mass index or fasting glucose levels (which are more precise than non-fasting glucose). These biomarkers are measured using standardized protocols and instruments, ensuring high accuracy in data collection and minimizing residual error.

Assume that the longitudinal marker $M_{ij}(t)$ takes the value $y_{ijk}$ within a given time interval $\left[t_{ijk},t_{ij(k+1)}\right)$ at time points $k=0,\ldots ,K_{ij}-1$ , with $t_{ijk}$ the time point for person j of twin pair i for which the marker is observed and define $t_{ij(k+1)}=t_{ij}$ , the observed survival time. Note that we typically do not have the marker value $y_{ijk}$ at $t_{ij}$ . Then the model for the conditional hazard $h_{ij}(t|v,x_{ij},y_{ij})$ for person j of twin pair i is given by^20^

$$h_{ij}(t|v_{i})=h_{0}(t)v_{i}\exp\;\left(\gamma x_{ij}+\alpha y_{ijk}\right)\;\text{for}\;t\in [t_{ijk},t_{ij(k+1)})$$

which is a standard Cox regression model with a time-dependent covariate.

For this model, the contribution of the i th twin to the conditional likelihood function $L_{i}(\alpha ,\gamma ,\xi |v_{i},x_{ij})$ is therefore^25^

(6) $$\begin{array}{rl} L_{i}(\alpha ,\gamma ,\xi |v_{i})= & \prod_{j=1}^{2}\prod_{k=0}^{K_{ij}-1}[h_{0}(t_{ij(k+1)})v_{i}\exp\;\left(\gamma x_{ij}+\alpha y_{ijk}\right){]}^{\delta_{ijk}}\\ & \times \exp\;\{-[H_{0}(t_{ij(k+1)})-H_{0}(t_{ijk})]v_{i}e^{\gamma x_{ij}+\alpha y_{ijk}}\} \end{array}$$

with $H_{0}(t)$ the cumulative baseline hazard function.

To obtain the marginal likelihood, we have to integrate over the distribution of the frailty $v_{i}$ .^4^ It is well known that due to the delayed entry, the unobserved frailties do not represent a random sample. Large frailties are underrepresented because these correspond to subjects who are more likely to experience the event early. To obtain the correct distribution, it is needed to condition the frailty distribution on cluster i being observed, that is we need to use the updated gamma frailty distribution. Let $g_{\theta }(v)$ be the distribution of the frailties v in the population. Now, using the updated frailty distribution, the log of the marginal likelihood can be formulated as follows:

(7) $$l\left(\alpha ,\gamma ,\xi ,\theta \right)=\sum_{i=1}^{N}\log\;\int_{v_{i}}L_{i}(\gamma ,\xi |v_{i},x_{ij})\;d\;G_{\theta }(v_{i}|t_{i1}>t_{i10},\ldots ,t_{in_{i}}>t_{in_{i}0})$$

Note that $g_{\theta }(v_{i}|t_{i1}>t_{i10},\ldots ,t_{in_{i}}>t_{in_{i}0})$ equals the distribution $\Gamma (\frac{1}{\theta },\frac{1}{\theta }+\sum_{j=1}^{n_{i}}H_{ij}(t_{ij0})e^{\gamma x_{ij}+\alpha y_{ij0}})$ .^26^ Further note that we assume that we observe only complete twin pairs. Maximum likelihood estimates of the parameters α , ξ , and θ can be obtained by maximizing this log-likelihood function.

This approach is called LOCF since at event time t for each individual still at risk the marker value $y_{ijk}$ with k such that $t_{ijk}<t$ and $t_{ij(k+1)}\geq t$ is used. When the assumption of constant marker values between the observed time points cannot be made, the estimate of α might be biased, and a regression calibration method should be used.

#### 2.2.2. Estimation of α by regression calibration methods

When it cannot be assumed that $y_{ijk}$ is constant between observed consecutive time points, we propose a two-step procedure where first a model for M(t) is obtained and then estimates for M(t) at a dense grid are plugged into the survival model. Such a method is available for singletons but not yet for clustered data. To use this approach for twin data, we propose a generalized linear mixed model for M(t) instead of simple linear regression. Using the observations on the covariate $y_{ijk}$ , we can first fit model (1) with $G_{ij}$ given in (2) to the data to obtain estimates ${\hat{\beta }}_{1}$ , $\Sigma_{b}$ , $\sigma_{u}^{2}$ , and $\sigma_{e}^{2}$ . Now, consider a dense grid $t_{0},\ldots ,t_{L-1}$ over the observed age range of the N twin pairs. Then for subject j of twin pair i , define $L_{ij}=\{l:t_{ij0}\leq t_{l}<t_{ij}\}$ , that is, all grid time points for which person j of twin pair i is observed. Thus, for all $l\in L_{ij}$ , we can obtain the following estimates:

(8) $${\hat{M}}_{ij}(t_{l})={\hat{\beta }}_{0}+{\hat{\beta }}_{1}t_{l}+{\hat{b}}_{ij0}+{\hat{b}}_{ij1}t+{\hat{u}}_{i}$$

with ${\hat{b}}_{ij0}$ , ${\hat{b}}_{ij1}$ , and ${\hat{u}}_{i}$ are the BLUP. Now, likelihood function (6) can be used to estimate α with index l instead of k and l running over $l\in L_{ij}$

(9) $$\begin{array}{rl} L_{i}(\alpha ,\gamma ,\xi |v_{i},x_{ij})= & \prod_{j=1}^{2}\prod_{l\in L_{ij}}[h_{0}(t_{\left(l+1\right)})v_{i}\exp\;\left(\gamma x_{ij}+\alpha {\hat{M}}_{ij}(t_{l})\right){]}^{\delta_{ijl}}\\ & \times \exp\;\{-[H_{0}(t_{\left(l+1\right)})-H_{0}(t_{l})]v_{i}e^{\gamma x_{ij}+\alpha {\hat{M}}_{ij}(t_{l})}\} \end{array}$$

To obtain the necessary estimates for equation (8), a model can be fitted using all data under the assumption that the marker profiles are not influenced by the dropout due to the occurrence of the event. This method is called ordinary regression calibration (ORC;^16–22^). If this assumption cannot be made, a way to relax it is to fit a model for each time point $t_{l}$ using only the subjects who have entered the study at time point $t_{l}$ , have not experienced the event yet, and are not yet censored (risk set regression calibration [RRC]). A drawback of this last method is that at the end of the study, the number of subjects at risk for the event might be small, and the error in estimation of ${\hat{M}}_{ij}(t_{l})$ might be large. Thus, a small number of individuals may violate the assumption of small $\sigma_{e}^{2}$ .

#### 2.2.3. Estimation of α by a joint likelihood approach

For M(t) endogenous, that is, $u_{i}$ represents unmeasured confounding linking M(t) and T , the estimator $\hat{\alpha }$ from the LOCF, ORC, or RCC approach might be biased. Maximizing the full likelihood function might be preferred. Unfortunately, this requires integration over the joint distribution of $u_{i}$ , $b_{i10}$ , $b_{i11}$ , $b_{i20}$ , and $b_{i21}$ , hence a computationally intensive numerical approximation over five-dimensional integrals in the survival model. However, if we assume that the model depicted in Figure 1(b) holds, that is, $Y_{i1}$ and $Y_{i2}$ contain all information on $b_{i10}$ , $b_{i11}$ , $b_{i20}$ , and $b_{i21}$ , we might plug in BLUP of $b_{i10}$ , $b_{i11}$ , $b_{i20}$ , and $b_{i21}$ and only perform numerical integration over $u_{i}$ . We denote this novel approach: two-stage JM. Specifically, just as in the calibration regression method, we first obtain estimates for $M_{ij}(t)$ for a dense grid of t , but then we compute

(10) $${\hat{G}}_{ij}(t_{k})={\hat{\beta }}_{0}+{\hat{\beta }}_{1}t_{k}+{\hat{b}}_{ij0}+{\hat{b}}_{ij1}t$$

that is, we do not plug in the estimate for $u_{i}$ . Next, we fit the following joint model:

(11) $$\begin{array}{rl} & Y_{ijk}={\hat{G}}_{ij}(t_{k})+u_{i}+\varepsilon_{ijk}={\hat{\beta }}_{0}+{\hat{\beta }}_{1}t_{k}+{\hat{b}}_{ij0}+{\hat{b}}_{ij1}t+u_{i}+\varepsilon_{ijk}\\ & h_{ij}(t|u_{i},v_{i},x_{ij})=h_{0}(t)v_{i}\exp\;\{\gamma x_{ij}+\alpha ({\hat{G}}_{ij}(t_{k})+u_{i}))\} \end{array}$$

Note that since this is a joint model, we do not need to assume that $\sigma_{e}^{2}$ is small. Thus, estimates of $\beta_{0}$ , $\beta_{1}$ , and $\sigma_{b}^{2}$ are obtained in the first step, while estimates of α , $\sigma_{u}^{2}$ , ξ , and $\sigma_{e}^{2}$ are obtained by maximizing the following joint model likelihood in the second step:

(12) $$\begin{array}{rl} \ell_{p}(\alpha ,\gamma ,\sigma_{u}^{2},\xi ,\sigma_{e}^{2})= & \sum_{i=1}^{N}\log\;\int_{u_{i}}[\int_{v_{i}}\prod_{j=1}^{2}{\tilde{L}}_{i,T}(\alpha ,\gamma ,\xi |u_{i},v_{i},x_{ij})\\ & \times g(v_{i}|t_{i1}>t_{i10}\cdots t_{in_{i}}>t_{in_{i}0})dv_{i}\prod_{j=1}^{2}{\tilde{L}}_{i,M}(\sigma_{e}^{2}|u_{i})]f_{u}(u_{i})du_{i} \end{array}$$

which involves just one integral over $u_{i}$ to be solved numerically. Here,

$$\begin{array}{rl} {\tilde{L}}_{i,T}(\alpha ,\gamma ,\xi |u_{i},v_{i},x_{ij})= & \prod_{j=1}^{2}[h_{0}(t_{ij})v_{i}\exp\;\left(\gamma x_{ij}+\alpha (\beta_{1}t+{\hat{b}}_{ij0}+{\hat{b}}_{ij1}t+u_{i})\right){]}^{\delta_{ij}}\\ & \times \exp\;\{-[H(t_{ij}|x_{ij},{\hat{M}}_{ij}(t|u_{i}))-H(t_{ij0}|x_{ij},{\hat{M}}_{ij}(t_{ij0}|u_{i}))]\} \end{array}$$

and

$${\tilde{L}}_{i,M}(\sigma_{b}^{2},\sigma_{e}^{2}|u_{i})=\prod_{j=1}^{2}\frac{1}{\sqrt{2\pi \sigma_{e}^{2}}}\exp\;\left(-\frac{(1-{\hat{\beta }}_{0}-{\hat{\beta }}_{1}t-{\hat{b}}_{ij0}-{\hat{b}}_{ij1}t-u_{i}{)}^{2}}{2\sigma_{e}^{2}}\right)$$

Note that this approach assumes that all confounding in the relation between T and Y , as well as the errors arising from using BLUPs, can be fully captured by $u_{i}$ , hence at the twin pair level and not at the individual level.

To summarize, for estimation of the effect of a longitudinal marker on a survival time in clusters subject to delayed entry, we have proposed four new methods, namely LOCF, ORC, RCC, and JM. The calibration methods ORC and RCC estimate the marker value for each individual for a grid of time points, either using one linear mixed model for all data (ORC) or by using separate models for each time point (RCC). The last two-stage method (JM) estimates only a part of the parameters of the model for $M_{ij}(t)$ in the first stage and then applies a joint model to estimate the variance of the random effect modeling the relationship between the longitudinal process and the survival outcome as well as the parameters of the survival model hence takes into account (partly) the randomness of $M_{ij}(t)$ .

### 2.3. Simulation design

We evaluate and compare the bias of the presented estimators across various approaches, considering both sparse and dense scenarios, and the presence or absence of measurements. To do so, we simulate from a joint model for longitudinal and time-to-event data. Suppose that there are G groups with $n_{i}$ individuals in the ith group, i=1,2,…,G and j=1,2 . Let $y_{ijk}=y_{ij}(t_{k})$ denote the response of subject j in cluster i at time $t_{ijl},l=1,\ldots ,n_{ij}$ . For simplicity, we do not consider a random slope for the longitudinal model. We simulate data to follow the following theoretical model:

(13) $$\begin{array}{rl} y_{ijk} & =M_{ij}(t_{k})+\varepsilon_{ijl}=1+b_{ij}+u_{i}+0.01t_{k}+\varepsilon_{ijk}\\ h_{ij}(t|b_{ij},u_{i},v_{i}) & =h_{0}(t)v_{i}\exp\;\{\alpha (1+b_{ij}+u_{i}+0.01t)\} \end{array}$$

where the random intercept $b_{ij}∼N(0,\sigma_{b}^{2})$ , the random cluster effect $u_{i}∼N(0,\sigma_{u}^{2}),$ and $\varepsilon_{ijk}∼N(0,\sigma_{e}^{2})$ . Assuming a Weibull baseline hazard $h_{0}(t)=\lambda \rho t^{\rho -1}$ , the cumulative hazard function is given by

$$H\left(t|M_{ij}(t),v_{i}\right)=\int_{0}^{t}\lambda \rho s^{\rho -1}v_{i}\exp\;\left(\alpha M_{ij}(s)\right)ds$$

Now, define the random variable W as a function of the random variable T as follows:

$$W=S\left(T|M_{ij}(T),v_{i}\right)\;=\;\exp\;\left[-H\left(T|M_{ij}(T),v_{i}\right)\right]$$

The random variable w is uniformly distributed U[0,1] . To generate survival times $t_{i1}$ and $t_{i2}$ , we sample a frailty $v_{i}$ from the gamma distributions and the random effects $b_{i1}$ , $b_{i2}$ , and $u_{i}$ from the normal distribution. Then we generate for each subject a w from the uniform distribution U(0,1) . Next, we can obtain a random value for T=t for each subject as follows:

(14) $$-\log\;(w)=H\left(t|M_{ij}(t),v_{i}\right)=\int_{0}^{t}\lambda \rho s^{\rho -1}v_{i}\exp\;(\alpha (\beta_{0}+b_{ij}+u_{i}+\beta_{1}s))\text{d}s$$

where numerical integration is used to find t .

We assume that right censoring follows a uniform distribution U[0,15] . Next, we simulate individual-specific entry time points $t_{ij0}$ as follows: a person has a probability of 0.5 to enter the study with a delay, that is, $t_{ij0}>0$ . If a person is delayed, we sample from the uniform distribution U(0,5) to obtain the truncation time $t_{ij0}$ . We then generate follow-up times $t_{ijk}$ , at points $k=0,\ldots ,K_{ij}-1$ for an individual such that $t_{ij0}\leq t_{ijk}.$ Then, for each j , we can compute $y_{ijk}$ at time points $t_{k}$ .

The following parameter values are used $\beta_{0}=1,\;\beta_{1}=0.01,\;\sigma_{b}^{2}=0.1^{2},\;\sigma_{u}^{2}=0.1^{2}$ , and frailty variance θ=0.5 . Finally, for the baseline hazard, we use a shape parameter ρ of 2 and a scale parameter λ of 0.001. The value of $\sigma_{\varepsilon }$ varies across the considered scenarios.

We report the relative bias (reBias) and standard deviation (SD) for estimation of parameters in 1000 Monte Carlo trials for LOCF, regression calibration, and joint model in the main text. Results for mean square error (MSE) and coverage probabilities (CPs) are given in the Appendix. All methods are fit on the same simulated datasets. Inc.G represents the number of clusters included in the analysis after truncation and after removal of singletons (i.e., we only consider fully observed clusters). For all models, we fit a Weibull proportional hazards model.

### 2.4. Simulation results

#### 2.4.1. Scenario 1: Dense and no measurement error

For this scenario, the longitudinal measurements are taken with a regular gap of 2 for all individuals, that is, at regular time points 0,2,4,6,… and the variance $\sigma_{\varepsilon }$ is equal to zero (no measurement error). Table 1 and Table 6 in the Appendix show the performance of LOCF and the “naive” method in estimating the parameters of the gamma shared frailty model in the presence of delayed entry and longitudinal markers. The naive method ignores delayed entry and appearsto give biased results, especially for larger α . The LOCF performed well in this setting.

#### 2.4.2. Scenario 2: Sparse and no measurement error

For this scenario, all individuals have three or fewer measurements (i.e., $n_{ij}\leq 3$ ) and there is no measurement error ( $\sigma_{\varepsilon }=0$ ). Here, we compare the performance of LOCF, RRC, and ORC.

**Table 1.** Simulation study to investigate the effect of the magnitude of the longitudinal marker effect α .

				Naive		LOCF	
α	Par.	Inc.G	Events	reBias (MCSE)	SD (MCSE)	reBias (MCSE)	SD (MCSE)
1	α	1637	578	− 0.029 (0.010)	0.300 (0.007)	− 0.022 (0.010)	0.301 (0.007)
	θ	1637	578	0.027 (0.011)	0.181 (0.004)	0.034 (0.011)	0.181 (0.004)
2	α	1584	1135	− 0.031 (0.004)	0.242 (0.005)	− 0.012 (0.004)	0.246 (0.006)
	θ	1584	1135	− 0.004 (0.006)	0.093 (0.002)	0.012 (0.006)	0.095 (0.002)
3	α	1465	1633	− 0.054 (0.003)	0.228 (0.005)	− 0.016 (0.003)	0.242 (0.005)
	θ	1465	1633	− 0.032 (0.004)	0.062 (0.001)	− 0.002 (0.004)	0.063 (0.001)

LOCF: last observation carried forward; reBias: relative bias; MCSE: Monte Carlo standard error; SD: standard deviation.

Naive is set up for modeling longitudinal markers without adjusting for delayed entry.

The results are depicted in Table 2 and in Table 7 in the Appendix. LOCF appeared to perform better for larger values of α . This can be explained by the data generation mechanism. Note that all individuals have ≤ three measurements during the study (not taken at regular time-points) and that we hold all other parameters fixed except for the value of α during the data generation. Generally, for larger α while holding other settings constant results in lower values of generated survival time, while smaller α results in larger values of generated survival time. Thus, we have less sparse measurements for larger α . Both α and the sparseness of measurements are changing in this case. Overall, the regression calibration approaches outperform the LOCF.

**Table 2.** Simulation study to investigate the effect of the magnitude of the longitudinal marker effect α .

				LOCF		RRC		ORC	
α	Par.	Inc.G	Events	reBias (MCSE)	SD (MCSE)	reBias (MCSE)	SD (MCSE)	reBias (MCSE)	SD (MCSE)
1	α	1637	577	− 0.306 (0.011)	0.342 (0.008)	− 0.031 (0.012)	0.365 (0.008)	0.040 (0.015)	0.466 (0.010)
	θ	1637	577	− 0.062 (0.010)	0.159 (0.004)	− 0.018 (0.011)	0.178 (0.004)	− 0.043 (0.011)	0.175 (0.004)
2	α	1584	1136	− 0.187 (0.004)	0.252 (0.006)	− 0.021 (0.005)	0.296 (0.007)	− 0.054 (0.004)	0.228 (0.005)
	θ	1584	1136	− 0.079 (0.006)	0.087 (0.002)	− 0.019 (0.006)	0.099 (0.002)	− 0.032 (0.004)	0.062 (0.001)
3	α	1464	1633	− 0.140 (0.002)	0.231 (0.005)	− 0.020 (0.003)	0.272 (0.006)	− 0.037 (0.004)	0.361 (0.008)
	θ	1464	1633	− 0.093 (0.004)	0.060 (0.001)	0.005 (0.004)	0.068 (0.002)	− 0.066 (0.004)	0.066 (0.002)

LOCF: last observation carried forward; RRC: risk set regression calibration; ORC: ordinary regression calibration; reBias: relative bias; MCSE: Monte Carlo standard error; SD: standard deviation.

**Table 3.** Simulation study to investigate the effect of the magnitude of the longitudinal marker effect α .

				LOCF		RRC		ORC		JM	
α	Par.	Inc.G	Events	reBias (MCSE)	SD (MCSE)	reBias (MCSE)	SD (MCSE)	reBias (MCSE)	SD (MCSE)	reBias (MCSE)	SD (MCSE)
1	α	1637	577	− 0.542 (0.008)	0.255 (0.006)	− 0.010 (0.016)	0.511 (0.011)	0.002 (0.015)	0.488 (0.011)	0.002 (0.011)	0.337 (0.008)
	θ	1637	577	− 0.034 (0.011)	0.172 (0.004)	0.085 (0.014)	0.221 (0.005)	− 0.076 (0.012)	0.189 (0.004)	− 0.013 (0.012)	0.175 (0.004)
2	α	1584	1136	− 0.493 (0.003)	0.200 (0.004)	− 0.009 (0.005)	0.345 (0.008)	− 0.033 (0.006)	0.371 (0.008)	0.005 (0.005)	0.287 (0.007)
	θ	1584	1136	− 0.077 (0.006)	0.092 (0.002)	0.014 (0.007)	0.103 (0.002)	− 0.053 (0.007)	0.107 (0.002)	− 0.003 (0.007)	0.093 (0.002)
3	α	1464	1633	− 0.494 (0.002)	0.164 (0.004)	− 0.035 (0.003)	0.306 (0.007)	− 0.078 (0.004)	0.381 (0.009)	− 0.010 (0.003)	0.287 (0.007)
	θ	1464	1633	− 0.109 (0.004)	0.063 (0.001)	− 0.006 (0.004)	0.067 (0.001)	− 0.048 (0.005)	0.073 (0.002)	− 0.022 (0.005)	0.062 (0.001)

LOCF: last observation carried forward; RRC: risk set regression calibration; ORC: ordinary regression calibration; reBias: relative bias; MCSE: Monte Carlo standard error; SD: standard deviation.

We let the $\sigma_{\varepsilon }=0.1$ .

**Table 4.** Simulation study to investigate the effect of the magnitude of measurement error $\sigma_{\varepsilon }^{2}$ .

				LOCF		RRC		ORC		JM	
$\sigma_{\varepsilon }$	Par.	Inc.G	Events	reBias (MCSE)	SD (MCSE)	reBias (MCSE)	SD (MCSE)	reBias (MCSE)	SD (MCSE)	reBias (MCSE)	SD (MCSE)
0	α	1584	1136	− 0.187 (0.004)	0.252 (0.006)	− 0.021 (0.005)	0.296 (0.007)	− 0.054 (0.004)	0.228 (0.005)		
	θ	1584	1136	− 0.079 (0.006)	0.087 (0.002)	− 0.019 (0.006)	0.099 (0.002)	− 0.032 (0.004)	0.062 (0.001)		
0.1	α	1584	1136	− 0.493 (0.003)	0.200 (0.004)	− 0.009 (0.005)	0.345 (0.008)	− 0.033 (0.006)	0.371 (0.008)	0.005 (0.005)	0.287 (0.007)
	θ	1584	1136	− 0.077 (0.006)	0.092 (0.002)	0.014 (0.007)	0.103 (0.002)	− 0.053 (0.007)	0.107 (0.002)	− 0.003 (0.006)	0.093 (0.002)
0.2	α	1584	1136	− 0.765 (0.002)	0.132 (0.003)	− 0.035 (0.007)	0.421 (0.009)	− 0.044 (0.007)	0.466 (0.010)	− 0.013 (0.005)	0.324 (0.008)
	θ	1584	1136	− 0.046 (0.006)	0.094 (0.002)	0.007 (0.007)	0.105 (0.002)	− 0.034 (0.006)	0.098 (0.002)	0.005 (0.006)	0.097 (0.002)
0.3	α	1584	1136	− 0.876 (0.001)	0.094 (0.002)	− 0.094 (0.008)	0.476 (0.011)	− 0.082 (0.009)	0.588 (0.013)	− 0.005 (0.005)	0.307 (0.007)
	θ	1584	1136	− 0.025 (0.006)	0.096 (0.002)	0.008 (0.007)	0.106 (0.002)	− 0.012 (0.006)	0.100 (0.002)	− 0.005 (0.006)	0.098 (0.002)
0.6	α	1584	1136	− 0.965 (0.001)	0.048 (0.001)	− 0.499 (0.009)	0.585 (0.013)	− 0.097 (0.020)	1.270 (0.028)	0.002 (0.006)	0.331 (0.008)
	θ	1584	1136	− 0.005 (0.006)	0.096 (0.002)	0.012 (0.007)	0.107 (0.002)	0.003 (0.006)	0.100 (0.002)	− 0.005 (0.006)	0.098 (0.002)

LOCF: last observation carried forward; RRC: risk set regression calibration; ORC: ordinary regression calibration reBias: relative bias; MCSE: Monte Carlo standard error; SD: standard deviation.

We let the α=2 .

**Table 5.** Parameter estimates assuming gamma frailty distribution and BMD as a longitudinal marker in the presence of delayed entry for only twin pairs (383 dizygotic twin pairs).

	LOCF	RRC	ORC	Joint model
Variable	Estimate (s.e.)	Estimate (s.e.)	Estimate (s.e.)	Estimate (s.e.)
exp(α)	0.081 (0.072)	0.054 (0.053)	0.033 (0.039)	0.018 (0.022)
θ	0.280 (0.291)	0.222 (0.294)	0.294 (0.291)	0.805 (0.372)
λ	<0.001 (0.001)	<0.001 (0.002)	0.001 (0.008)	0.003 (0.018)
ρ	2.579 (1.099)	2.394 (1.178)	2.244 (1.095)	2.292 (1.072)
$\sigma_{u}$				0.040 (0.002)
$\sigma_{e}$				0.071 (0.001)

BMD: bone mineral density; LOCF: last observation carried forward; RRC: risk set regression calibration; ORC: ordinary regression calibration; s.e.: standard error.

The number of observed events is 138. ID-level random slope included in the model.

#### 2.4.3. Scenario 3: Sparse with measurement error

The simulations performed using Scenarios 1 and 2 assume that M(t) is measured without an error. We now consider the scenario of measurement error. Similar to Scenario 2, we consider all individuals to have ≤3 measurements during the study.

We perform simulations to investigate the effect of the magnitude of measurement error $\sigma_{\varepsilon }^{2}$ and of the longitudinal marker effect α on the performance of the various estimators of α and θ .

Tables 3 and 4, along with Tables 8 and 9 in the Appendix, present the performance of the proposed methods (LOCF, RRC, ORC, and JM) in estimating the parameters of the gamma shared frailty model under delayed entry, for various values of α and $\sigma_{e}^{2}$ , respectively.

When using the LOCF approach, the effect α of the longitudinal marker is estimated with a negative relative bias in all scenarios (i.e., for various magnitudes of α or of the measurement error $\sigma_{\varepsilon }^{2}$ ). The LOCF estimator for α has a largest negative bias of about 54% when α = 1 (Table 3 and of about 96% when $\sigma_{\varepsilon }$ = 0.6, see Table 4).

The two regression calibration approaches yield similar results, except for cases of a large magnitude of α ( α=3 ), and of a large magnitude of the measurement error ( $\sigma_{\varepsilon }=0.6$ ). For a large magnitude of the effect of the longitudinal marker ( α =3), RRC performs better than ORC. From Table 3, the largest bias in the RRC estimator of α is about 3%, while the largest bias in the ORC estimator of α is about 8%. For a large magnitude of measurement error ( $\sigma_{\varepsilon }=0.6$ ), ORC performs better than RRC. From Table 4, the RRC estimator of α has the largest bias of about 50% while the ORC estimator of α has a bias <10% for the considered scenarios.

The JM approach performs well in estimating the effect α of the longitudinal marker for all magnitudes considered of the longitudinal marker effect α and of the measurement error $\sigma_{\varepsilon }^{2}$ . From Table 3, the joint model estimator of α has the largest bias of about 5% when α=1 , while from Table 4, this estimator of α has the largest bias of about 6% for large $\sigma_{\varepsilon }$ .

All the methods appear to estimate θ with low bias for all considered magnitudes of the effect α and of the measurement error $\sigma_{\varepsilon }^{2}$ (largest bias for LOCF estimator of θ is about 10% when α=3 , the largest bias for the calibration estimators of θ is about 8% when α=1 and the largest bias for the JM estimator of θ is about 3% when α=3 as shown in Table 3). Results on performance in estimation $\sigma_{u}$ when fitting the joint model are given in the Appendix (see Tables 10 and 11) for various values of the longitudinal marker effect α and of the measurement error $\sigma_{\varepsilon }^{2}$ .

Overall, the calibration and the JM approaches yield less bias in the estimation of parameters as compared to the LOCF approach in all considered scenarios. The bias in the estimation of α increases when the measurement error increases, with the exception of the JM estimator. Both LOCF and the two regression calibration methods result in consistent underestimation of α , the effect of the longitudinal marker. This underestimation is more severe for the LOCF approach. It appears that in the considered scenarios, the ORC performs well in estimation of the effect of the longitudinal marker, even for large measurement error, as it yields notably low bias.

The joint model had convergence issues (about 10% of the simulated datasets do not yield standard error estimates of the parameter α ). In terms of computational time, the Joint Model (JM) was the most time-consuming. For example, in scenario 3, when α =2, JM required on average 190 minutes per simulation run. In contrast, the RRC, ORC, and LOCF approaches were substantially faster, taking approximately 1 min 40 s, 1 min 10 s, and 18 s, respectively.

## 3. Application: The effect of BMD on fracture incidence

We have access to longitudinal BMD observations and age at fractures of female dizygotic twins of 50 years of age and older from the TwinsUK (https://twinsuk.ac.uk). In Muli et al.,^23^ this dataset was analyzed using BMD as a time-fixed covariate to estimate the probability of a fracture in the next time period, that is, only the BMD at entry was used. BMD at entry appeared to be a statistically significant risk factor for fracture incidence. Here, we aim to estimate the relationship between BMD and fracture incidence over age. A joint model might be appropriate when there are genetic factors influencing both the BMD outcome over time and fracture incidence. On the other hand, the heritability of fracture incidence is lower, and identified genetic loci also have an effect on BMD, hence only a direct effect of BMD on fracture incidence might be biologically plausible as well.^27^ Since the twins enter the study at different ages, we need to use the approaches developed in this paper. For all approaches, we consider a Weibull baseline hazard and gamma frailty distribution.

**Figure 2.**
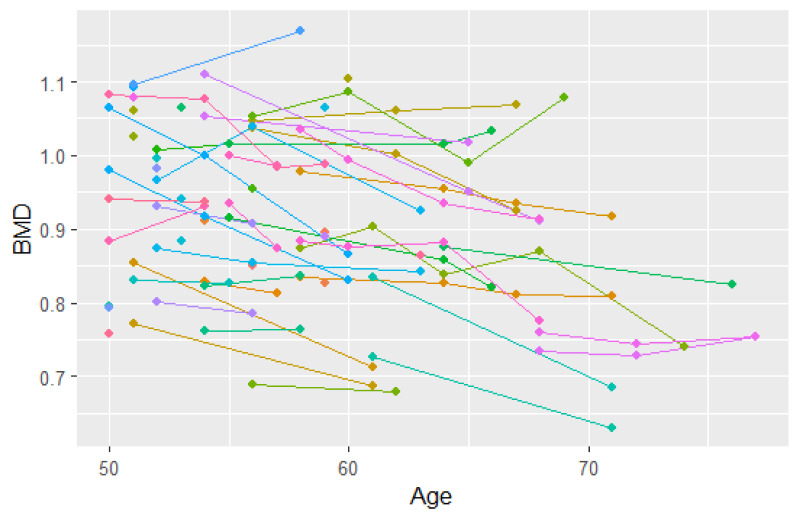
Spaghetti plot for 50 participants (a subset of the data).

**Figure 3.**
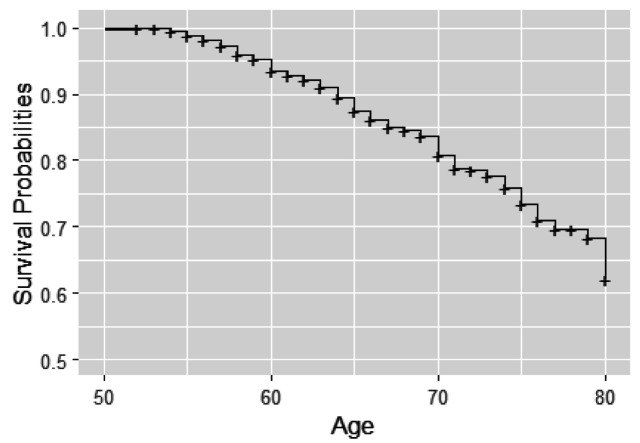
Kaplan–Meier curve for fractures using age as time scale.

In this analysis, we consider BMD measurements after age 50 for the 766 individuals from 383 twin pairs (for results of the analysis of 383 twin pairs and 262 single twins see Table 12). For a sample of twins, their BMD profiles over age are given in Figure 2. We observe that indeed subjects enter the study at different ages and that for most subjects, BMD decreases with age. Furthermore, we notice that the time gaps between observed time points are quite large. The Kaplan–Meier curve taking into account delayed entry is depicted in Figure 3. It appeared that the probability of having a fracture before the age of 80 years is 0.3 to 0.4 in this cohort. Analysis of the longitudinal observed BMD showed that a model with a random intercept and slope at the individual level and a random intercept at the twin-level fitted the data well. All three random factors appeared to be statistically significant.

When modeling the BMD over time, we used mixed models including a random intercept and slope to model the correlation over time for a subject and a shared twin effect to model the correlation between twins. For ORC, we fit such a single mixed effects model in the first stage using all available data to compute predicted covariate values for each subject at each age in the sample. For RRC, we split the time into intervals: there are 30 unique ages at entry/event times (minimum age at entry is 50 years, minimum event time is 52 years, while the maximum event time is 85 years). For ages 51 and 52, we could only model a random twin effect, because individuals do not have repeated measurements at those time points. For the other unique age at entry/event times, the same mixed model as for ORC was used. From these models, we compute the predicted value of the covariate for individuals still at risk at a specific event time. For ORC and RRC, the data are arranged in start–stop format and the shared frailty model by maximizing the likelihood function (7). For the JM approach, we used the mixed model of the first step of ORC to obtain estimates of the fixed parameters and the empirical Bayes estimates of the random intercepts and slopes. Then, model (11) is fitted using the likelihood function (12).

All approaches gave a highly significant effect of BMD on fracture incidence (Table 5) ( p -values of <0.001 ). RRC, ORC, and JM gave stronger (smaller) point estimates as compared to the LOCF. JM provided the strongest (smallest) effect estimate (0.018, s.e.: 0.022). Concerning the variance of the frailty ( θ ), the estimates vary from 0.222 (RRC) to 0.805 (JM). The residual variance of the mixed model was quite small ( $\sigma_{e}=0.071$ ).

In a separate analysis, we model the effect of BMD on fracture incidence in the monozygotic dataset, using BMD measurements taken after age 50. This analysis includes 288 monozygotic twin pairs and 188 monozygotic single twins (see Tables 13 and 14 in the Appendix).

## 4. Discussion

In this paper, we propose a novel joint model for a longitudinal marker and a survival outcome in twin studies. We developed four approaches (LOCF, ORC, RRC, and JM) to estimate the relationship between a longitudinal marker and a survival outcome, and the frailty variance using data from twins who enter the study at different ages. Through simulations, we showed that LOCF performs well for a dense grid of observed marker values. ORC, RCC, and JM also perform well for a sparse grid. When the measurement error is large ( $\sigma_{e}\geq 0.6$ ), ORC and RCC provided bias results while JM still performed well.

Our novel JM approach can be interpreted as a hybrid approach in between a full joint modeling approach and the regression calibration approach. The advantage of a full JM approach is that it captures all variation that might be present in the data. Unfortunately, it is typically computationally infeasible to fit this model to the data. In contrast, our JM approach is computationally feasible, while regression calibration methods such as ORC and RRC are even more computationally efficient. Therefore, depending on the type of longitudinal marker, the density of the observations over time, and the magnitude of the measurement error, the calibration methods might be preferred. Specifically, in our simulation study, LOCF appears to perform well with small measurement error and dense grids, making it the standard method, as it is for singletons, for this scenario. ORC and RRC appear to be the preferred options for sparse grids with low measurement error. Low measurement error is a reasonable assumption in many practical settings. In studies in which rigorous data collection procedures are followed with standardized protocols, measurement error can be minimized. For example, in longitudinal studies measuring biomarkers such as blood glucose levels, where devices are regularly calibrated, the measurement error tends to be small. However, in studies with less controlled data collection processes, one should expect a larger measurement error, and in these cases, the joint model approach might be preferable. On the other hand, with a large measurement error, JM might show numerical instability. In such cases, ORC is an acceptable alternative.

The methods were applied to real data from the TwinUK twin registry. Here, LOCF yielded a weaker estimate than the other methods. Indeed, the gaps between the observed time points are quite large; hence, the two-stage approach should perform better. For the JM approach, the estimate of the variance of the frailty was larger than for the calibration methods. The differences between the estimates of the effect of BMD and of the frailty variance across the approaches are not statistically significant. The standard errors for the effect of BMD on survival are similar, although they slightly increase for the more advanced methods (ORC and JM). This can be expected, since the more advanced methods better capture the randomness in the longitudinal marker. For a full joint modeling approach, these standard errors might increase even further. Unfortunately, it appears computationally infeasible to apply this method to this dataset. However, given the small differences in standard errors between the methods used, a full JM approach would likely yield the same conclusion.

Our method is relevant for other survival outcomes and other twin studies beyond TwinsUK. Using age as an underlying time variable is often better interpretable than arbitrary follow-up time and results in more parsimonious models. For example, Cirulli et al.^1^ modeled the effect of metabolome health on cardiovascular events in TwinsUK. They used follow-up as the underlying time variable and age-at-entry as covariate in a Cox proportional hazard model. For Danish twins, Tan et al.^2^ modeled the effect of DNA methylation on mortality, adjusting for age at blood sampling. Also, when combining different studies in a meta-analysis, typically, follow-up times across studies are not comparable, and using age would be more appropriate. Our methods allow for the utilization of age as an underlying time scale, providing a more appropriate approach for analyzing longitudinal markers in twin studies. Furthermore, our proposed methods could be applied to other types of clustered survival data, such as paired data involving organs such as glycoma onset in eyes and hip replacement in hips due to osteoarthritis.

We proposed a novel two-stage approach for fitting a joint model as an alternative for a full joint estimation of all the parameters. We hypothesize that the computational complexity of a full-likelihood approach is often unfeasible in applications. Our two-stage joint model seems to represent a good tradeoff between complexity and applicability in the presence of measurement error. We have chosen to jointly model the cluster-specific parameters and the random error term of the longitudinal marker process and take the subject-specific random effects of the longitudinal marker process as independent of the time-to-event process. This assumption aligns with the idea that in twin studies, the shared unmeasured confounding between the biomarker and survival outcome is more likely to operate through the twin-level effect, rather than individual-specific effects, which justifies the focus on modeling $u_{i}$ while using BLUPs for the individual-specific deviations.

Several extensions of the methods can be considered, namely, modeling the recurrent fracture incidence and a more complex within-cluster structure to also include monozygotic twins. Additionally, we considered only complete twin pairs in our analysis, with results for including incomplete twin pairs provided in the Appendix. However, the likelihood function used is conditioned on both twins entering the study, which is violated when including single-twin members, as previously discussed in the context of time-fixed covariates.^28^ Instead, one should condition on the entry of one twin, which complicates the likelihood function. Furthermore, to account for dropouts due to severe illness and death, competing risk models might be considered. Another extension is to use both the calendar and age scale simultaneously as proposed by Bower et al.^29^ Lastly, we considered a Weibull baseline hazard in the simulation study and data application because it is commonly used as it is computationally attractive, and can take different shapes depending on the value of the shape parameter. It is straightforward to use more flexible baseline hazards, for example, by using splines.^30^

In conclusion, the study of longitudinal markers in the presence of clustered survival data and delayed entry necessitates careful consideration of model choice. We have introduced a set of new models and estimation methods for this research area, along with specific recommendations based on the presumed underlying relationships, observation density, and error structures.

## Appendix

**Table 6.** Simulation study to investigate the effect of the magnitude of the longitudinal marker effect α .

				Naive		LOCF	
α	Par.	Inc.G	Events	MSE	CP	MSE	CP
1	α	1637	578	0.091	0.962	0.091	0.965
	θ	1637	578	0.033	0.957	0.033	0.960
2	α	1584	1135	0.062	0.954	0.061	0.948
	θ	1584	1135	0.009	0.949	0.009	0.948
3	α	1465	1633	0.078	0.896	0.060	0.940
	θ	1465	1633	0.004	0.939	0.004	0.950

LOCF: last observation carried forward; MSE: mean square error; CP: coverage probability.

Naive is set up for modeling longitudinal markers without adjusting for delayed entry.

**Table 7.** Simulation study to investigate the effect of the magnitude of the longitudinal marker effect α .

				LOCF		RRC		ORC	
α	Par.	Inc.G	Events	MSE	CP	MSE	CP	MSE	CP
1	α	1637	577	0.211	0.805	0.134	0.933	0.218	0.951
	θ	1637	577	0.026	0.953	0.032	0.957	0.031	0.955
2	α	1584	1136	0.203	0.675	0.089	0.950	0.078	0.896
	θ	1584	1136	0.009	0.915	0.010	0.930	0.004	0.939
3	α	1464	1633	0.229	0.565	0.077	0.965	0.143	0.929
	θ	1464	1633	0.006	0.868	0.005	0.950	0.005	0.929

LOCF: last observation carried forward; RRC: risk set regression calibration; ORC: ordinary regression calibration; MSE: mean square error; CP: coverage probability.

**Table 8.** Simulation study to investigate the effect of the magnitude of the longitudinal marker effect α .

				LOCF		RRC		ORC		JM	
α	Par.	Inc.G	Events	MSE	CP	MSE	CP	MSE	CP	MSE	CP
1	α	1637	577	0.359	0.455	0.256	0.920	0.236	0.956	0.113	0.923
	θ	1637	577	0.030	0.940	0.049	0.880	0.037	0.917	0.031	0.978
2	α	1584	1136	1.014	0.000	0.118	0.921	0.141	0.965	0.082	0.900
	θ	1584	1136	0.010	0.910	0.011	0.978	0.012	0.930	0.009	0.991
3	α	1464	1633	2.222	0.000	0.104	0.939	0.199	0.900	0.083	0.900
	θ	1464	1633	0.007	0.810	0.004	0.945	0.006	0.905	0.004	0.994

LOCF: last observation carried forward; RRC: risk set regression calibration; ORC: ordinary regression calibration; JM: joint modeling; MSE: mean square error; CP: coverage probability.

We let the $\sigma_{\varepsilon }=0.1$ .

**Table 9.** Simulation study to investigate the effect of the magnitude of measurement error $\sigma_{\varepsilon }^{2}$ .

				LOCF		RRC		ORC		JM	
$\sigma_{\varepsilon }$	Par.	Inc.G	Events	MSE	CP	MSE	CP	MSE	CP	MSE	CP
0	α	1584	1136	0.203	0.675	0.089	0.950	0.078	0.896		
	θ	1584	1136	0.009	0.915	0.010	0.930	0.004	0.939		
0.1	α	1584	1136	1.014	0.000	0.118	0.921	0.141	0.965	0.082	0.900
	θ	1584	1136	0.010	0.910	0.011	0.978	0.012	0.930	0.009	0.991
0.2	α	1584	1136	2.359	0.000	0.181	0.920	0.224	0.955	0.105	0.929
	θ	1584	1136	0.009	0.920	0.011	0.950	0.010	0.950	0.009	0.991
0.3	α	1584	1136	3.081	0.000	0.260	0.947	0.371	0.915	0.094	0.956
	θ	1584	1136	0.009	0.930	0.011	0.940	0.010	0.960	0.010	0.994
0.6	α	1584	1136	3.730	0.000	1.334	0.562	1.642	0.915	0.109	0.940
	θ	1584	1136	0.009	0.945	0.011	0.927	0.010	0.940	0.010	0.999

LOCF: last observation carried forward; RRC: risk set regression calibration; ORC: ordinary regression calibration; JM: joint modeling; MSE: mean square error; CP: coverage probability.

We let the α=2 .

**Table 10.** Estimation of $\sigma_{u}$ : Effect of magnitude of longitudinal marker effect α .

	JM	
α	reBias (MCSE)	SD (MCSE)
1	− 0.180 (0.001)	0.004 (0.000)
2	− 0.182 (0.001)	0.004 (0.000)
3	− 0.187 (0.001)	0.004 (0.000)

JM: joint modeling; reBias: relative bias; MCSE: Monte Carlo standard error; SD: standard deviation.

**Table 11.** Estimation of $\sigma_{u}$ : Effect of magnitude of measurement error $\sigma_{\varepsilon }^{2}$ .

	JM	
$\sigma_{\varepsilon }$	reBias (MCSE)	SD (MCSE)
0.1	− 0.182 (0.001)	0.004 (0.000)
0.2	− 0.184 (0.001)	0.007 (0.000)
0.3	− 0.193 (0.001)	0.013 (0.000)
0.6	− 0.150 (0.001)	0.025 (0.001)

JM: joint modeling; reBias: relative bias; MCSE: Monte Carlo standard error; SD: standard deviation.

**Table 12.** Parameter estimates assuming gamma frailty distribution and BMD as a longitudinal marker in the presence of delayed entry for 383 dizygotic twin pairs and 262 dizygotic single twins.

	LOCF	RRC	ORC	Joint model
Variable	Estimate (s.e.)	Estimate (s.e.)	Estimate (s.e.)	Estimate (s.e.)
exp(α)	0.055 (0.042)	0.039 (0.033)	0.025 (0.025)	0.017 (0.009)
θ	0.123 (0.212)	0.045 (0.179)	0.109 (0.210)	0.336 (0.077)
λ	0.003 (0.014)	0.023 (0.113)	0.045 (0.227)	0.010 (0.007)
ρ	1.944 (0.988)	1.568 (1.069)	1.522 (1.047)	1.911 (0.130)
$\sigma_{u}$				0.040 (0.002)
$\sigma_{e}$				0.067 (0.001)

BMD: bone mineral density; LOCF: last observation carried forward; RRC: risk set regression calibration; ORC: ordinary regression calibration; s.e.: standard error.

The number of observed events is 170. Id-level random slope included in the model.

**Table 13.** Parameter estimates assuming gamma frailty distribution and BMD as a longitudinal marker in the presence of delayed entry for 288 monozygotic twin pairs.

	LOCF	RRC	ORC	Joint model
Variable	Estimate (s.e.)	Estimate (s.e.)	Estimate (s.e.)	Estimate (s.e.)
exp(α)	0.024 (0.038)	0.012 (0.021)	0.009 (0.017)	0.009 (0.000)
θ	1.366 (0.499)	1.363 (0.505)	1.390 (0.528)	0.090 (0.000)
λ	<0.001 (0.000)	<0.001 (0.000)	<0.001 (0.000)	0.050 (0.000)
ρ	5.804 (0.655)	5.673 (0.733)	5.480 (0.858)	2.343 (0.003)
$\sigma_{u}$				0.089 (0.000)
$\sigma_{e}$				0.058 (0.000)

BMD: bone mineral density; LOCF: last observation carried forward; RRC: risk set regression calibration; ORC: ordinary regression calibration; s.e.: standard error.

The number of observed events is 69.

**Table 14.** Parameter estimates assuming gamma frailty distribution and BMD as a longitudinal marker in the presence of delayed entry for 288 monozygotic twin pairs and 178 monozygotic singletons.

	LOCF	RRC	ORC	Joint model
Variable	Estimate (s.e.)	Estimate (s.e.)	Estimate (s.e.)	Estimate (s.e.)
exp(α)	0.040 (0.061)	0.037 (0.062)	0.032 (0.057)	0.024 (0.000)
θ	1.452 (0.741)	1.516 (0.775)	1.502 (0.784)	0.064 (0.000)
λ	<0.001 (0.000)	<0.001 (0.000)	<0.001 (0.000)	0.010 (0.000)
ρ	4.671 (1.499)	4.664 (1.577)	4.539 (1.615)	2.358 (0.004)
$\sigma_{u}$				0.077 (0.000)
$\sigma_{e}$				0.053 (0.000)

BMD: bone mineral density; LOCF: last observation carried forward; RRC: risk set regression calibration; ORC: ordinary regression calibration; s.e.: standard error.

The number of observed events is 91.
